# Supplementary material for: Application value of systemic immune-inflammation index in predicting severe Mycoplasma pneumoniae pneumonia
Source: PLoS One. 2025 Oct 9;20(10):e0333267. doi: 10.1371/journal.pone.0333267 (PMC12510598; doi:10.1371/journal.pone.0333267)
Supplement: Attachment — Submitted filename: Response to Reviewers.docx [file pone.0333267.s003.docx]

**PONE-D-25-26564**

Title:**Application value of Systemic Immune-Inflammation Index in predicting severe Mycoplasma pneumoniae pneumonia**

Dear editors in chief and reviewers:

Thank you very much for your letter and comments concerning our manuscript. All those comments are valuable and very helpful for improving our paper. We have studied comments carefully and have made correction which we hope meet with approval. Two copies of our manuscript with and without all revisions have been uploaded. The main corrections in the paper and our point-by-point responses to the reviewer’s comments are given below.

We extend our profound gratitude to the reviewers for your insightful and constructive suggestions,which have strengthened the quality and rigor of our manuscript.We hope that the revised manuscript has adequately addressed reviewers’ concerns and would be suitable for publication. We look forward to hearing from you regarding the suitability of the manuscript for publication in ***PLOS ONE***.

Thank you and best regards.

Xiaoya Xu,Corresponding author

Department of Neurology,Zigong First People's Hospital,

No.42, 1st Branch Road, Shangyi, Ziliujing District,

Zigong 643000, Sichuan Province, China

**Tel:** 86-0813-2121099

**Email:** [xxy820122@163.com](mailto:xxy820122@163.com)

****Responses to Academic Editor Comments****

1. ****In the title, the terms "novel inflammatory biomarkers" and "severe mycoplasma infections" are problematic. The reported biomarkers are not novel. The severity of mycoplasma infections is not defined as a pathological condition in the text.****

****Response:****

Thank you for your constructive comments.**We apologize for the inaccuracy in the title. We have revised the title to remove the term "novel," as the biomarkers (e.g., SII, SIRI) are indeed established in existing literature.**Title updated to: "Application value of Systemic Immune-Inflammation Index in predicting severe Mycoplasma pneumoniae pneumonia".

**Additionally, we have explicitly defined "severe mycoplasma infections" in the Introduction section, referencing clinical criteria (e.g., presence of respiratory failure, extrapulmonary complications, or requirement for intensive care) to clarify the pathological condition.**(see Page 6,Line 99–115, Revised Manuscript with Track Changes)

2. **Is serum amyloid A (SAA) determination a routine laboratory investigation?**

**Response:**

**Thank you for your question regarding the routine status of serum amyloid A (SAA) determination.In our clinical setting, SAA is routinely included in the panel of inflammatory markers for patients presenting with suspected infectious diseases—including pneumonia— as part of their initial laboratory workup. This practice aligns with the clinical workflow in our institution for evaluating and monitoring inflammatory responses in such patient populations**

3. **Subgrouping of patients was based on which criteria?**

****Response:****

Thank you for your insightful comment, which helps clarify an important methodological detail of our study.Patients in this study were subgrouped into two categories: "severe Mycoplasma pneumoniae pneumonia (MPP)" and "non-severe Mycoplasma pneumoniae pneumonia (MPP)". This subgrouping was strictly based on the clinical criteria specified in the Evidence-based guideline for the diagnosis and treatment of Mycoplasma pneumoniae pneumonia in children (2023).To ensure transparency and reproducibility, we have supplemented the key content of these criteria in both the Introduction and the Methods section (where we described the study’s patient grouping strategy) of the revised manuscript.(see Page 6,Line 99–115 and Page 7,Line 125–127）

4. **The authors extracted data from records, but why and how did they conduct interviews? Verbal consent was unacceptable.**

****Response:****

We apologize for the confusion. This was an error in the original manuscript: no interviews were conducted for this retrospective study. All data were extracted from electronic medical records. The reference to "verbal consent" was also incorrect and has been removed. For retrospective studies using de-identified data(in line with our study design), our institutional ethics board waived the requirement for informed consent, which is now clarified in the Ethics Statement. (see Page 7 ,line 135–139）

We deeply regret the inaccuracies in the original text and have double-checked the Methods and Ethics sections to ensure all content aligns with the actual study procedures.

5. **Line 146: The "S-W test" – do you mean the Shapiro-Wilk test?**

****Response:****

Yes, "S-W test" refers to the Shapiro-Wilk test. We have corrected the abbreviation to "Shapiro-Wilk test" at its first mention and added the abbreviation in parentheses for clarity. (see Page 8,Line 151)

6. **Table 1 (footnote: mean +/- SD), but results are presented as median (IQR). Day stay in hospital is the same for severe or non-severe infection – how?**

****Response:****

We sincerely apologize for the inconsistency in Table 1. The footnote has been corrected to "median (IQR)" to match the data presentation.(see Page 10, Line 174-175,table1).

Regarding hospital stay, the original analysis showed no statistically significant difference between groups, which is now clarified in the text with a p-value (p = 0.123) and a brief explanation that variability in individual recovery trajectories may account for this finding.There is partial overlap in the interquartile ranges of hospital stay between the two groups (non-severe:4–7days; severe:5–8 days), indicating considerable individual variability in hospital stay within both groups. This variability may be attributed to factors such as rapid treatment response in some severe cases and the presence of comorbidities or slower recovery subgroups among non-severe cases, which collectively resulted in no statistically significant difference in the overall comparison.

7. **Table 2 needs reanalysis to show different models. It is necessary to add beta-coefficients. A Cohen's test is needed to show the impact of sample size.**

****Response:****

Thank you for these constructive comments.We have reanalyzed Table 2 in accordance with your comments, and the relevant revisions have been reflected in the text.(see Page 11-12, Line 192-196,table2).

8. **Add plots of AUCs.**

****Response:****

Thank you for this insightful comment. As requested, we have supplemented the plots of the AUCs as Figure 2 in the revised manuscript. Additionally, to enhance the transparency of our study design, we have also added a flowchart illustrating the participant selection and exclusion criteria as Figure 1. In accordance with the journal's requirements, these figures have been uploaded as separate files.(see Figures1 and Figure 2 )

9. **References: Recheck the typing of the references.**

****Response:****

Thank you for this helpful suggestion. We have carefully reviewed all references to correct typographical errors. Specifically, we have addressed formatting issues in Reference 12 (misspelled journal name) and Reference 23 (incorrect DOI), ensuring compliance with the citation guidelines of PLOS ONE.(see Page 16–22, references )

**Responses to Journal Requirements**

**1. Ensure the manuscript meets PLOS ONE style requirements, including file naming.**

****Response:****

Thank you for this helpful reminder.The manuscript has been revised to adhere to PLOS ONE style guidelines, including title formatting, author affiliations, and section organization. All files are named according to the requirements (e.g., "Manuscript.pdf," "Response to Reviewers.pdf").

**2. Provide a validated ORCID iD for the corresponding author in Editorial Manager.**

****Response:****

Thank you for bringing this oversight to our attention. The corresponding author’s ORCID iD (0000-0003-2645-8637) has been successfully validated and submitted in Editorial Manager in accordance with your instructions.

**3.Address overlapping text with previous publications by citing sources or rephrasing.**

****Response:****

Thank you for this important suggestion. We have carefully revised all instances of overlapping text identified—either by rephrasing content to ensure original expression or by adding proper, contextually accurate citations to attribute the relevant previous publications. These revisions have been verified to ensure the text similarity rate fully complies with academic integrity standards. All such modifications are clearly highlighted in red text in the marked-up version of the manuscript for your easy reference.

**4. Clarify the role of funders in the study.**

****Response:****

Thank you for bringing this point to our attention. We have revised the funder statement to explicitly clarify the role of the funders in the study, and the updated content reads as follows: "This work was supported in part by the Zigong Science and Technology Bureau (Grant No. 2023YKYXT06). The funds were specifically allocated for researcher training in scientific competencies; however, the funders had no role in study design, data collection and analysis, the decision to publish, or the preparation of the manuscript." This revised funder statement has been included in the cover letter for your reference.

5. **Make all underlying data available per PLOS data policy.**

****Response:****

Thank you for reminding us of this requirement related to PLOS’s data policy.All non-identifiable underlying data supporting the findings of this study have been prepared as supplementary files and uploaded alongside the revised manuscript. These data comply with research ethics regulations and privacy protections, ensuring no sensitive patient information is included. A revised data availability statement has been added to the manuscript to clarify that the underlying data are available as supplementary materials within the submission.

**Responses to Reviewer #2 Comments**

**Comment 1: Clarify the inconsistency between "retrospective chart review" and mentions of "verbal informed consent/interviews."**

****Response:****

We sincerely apologize for the confusion caused by this inconsistency in the original manuscript. As noted, this was an error in the initial writing: the study design is strictly a retrospective chart review, and all data were extracted solely from electronic medical records. There were no interviews conducted in this study, so the reference to "verbal informed consent" was incorrect and has been completely removed from the revised manuscript.

Additionally, for retrospective studies utilizing de-identified data (such as this one), our institutional ethics board (IEB) has formally waived the requirement for informed consent. This information has been supplemented and clarified in the "Ethics Statement" section of the manuscript for full transparency (see Page 7, Lines 135–139). We appreciate you pointing out this oversight, as it has helped us improve the accuracy of the study’s methodological description

**Comment 2: Define each abbreviation at first use.**

****Response:****

Thank you for highlighting this important point regarding abbreviation clarity. We have carefully revised the entire manuscript to ensure every abbreviation is explicitly defined the first time it appears in the text.

**Comment 3: Avoid repetition (e.g., severity and complications of SMPP discussed multiple times).**

****Response:****

Thank you for identifying this issue of repetitive content—it has helped us refine the manuscript’s conciseness. We have carefully reviewed the entire text, with specific attention to the sections previously discussing the severity and complications of severe mycoplasma pneumoniae pneumonia (SMPP) .

We have removed redundant repetitive descriptions to ensure the manuscript is concise, aiming to enhance readability for readers.

**Comment 4:Add a flowchart of participant selection and exclusion criteria.**

****Response:****
Thank you for this constructive suggestion.We have added a flowchart of participant selection and exclusion criteria as figure 1.

**Comment 5: Lines 205–213 in the Discussion lack literature references – please add.**

****Response:****

Thank you for this valuable suggestion. We have added relevant citations to support the statements.

**Comment 6: Include a formal data availability statement.**

****Response:****

Thank you for reminding us of this requirement related to PLOS’s data policy.All non-identifiable underlying data supporting the findings of this study have been prepared as supplementary files and uploaded alongside the revised manuscript. These data comply with research ethics regulations and privacy protections, ensuring no sensitive patient information is included. A revised data availability statement has been added to the manuscript to clarify that the underlying data are available as supplementary materials within the submission.

**Comment 7: Improve language clarity and consistency.**

****Response:****

Thank you for highlighting this important aspect of the manuscript—enhancing language clarity and consistency is critical for ensuring our research is accurately understood by readers. We have taken comprehensive measures to address this: the entire manuscript has been revised and polished by a professional language editing team composed of native English speakers, with a focused effort on clarifying ambiguous sentences and correcting minor grammatical errors, awkward phrasing, and non-standard academic expressions.
